# Kinase Activity of PAR1b, Which Mediates Nuclear Translocation of the BRCA1 Tumor Suppressor, Is Potentiated by Nucleic Acid-Mediated PAR1b Multimerization

**DOI:** 10.3390/ijms23126634

**Published:** 2022-06-14

**Authors:** Hiroko Nishikawa, Priscillia Christiany, Takeru Hayashi, Hisashi Iizasa, Hironori Yoshiyama, Masanori Hatakeyama

**Affiliations:** 1 Division of Microbiology, Graduate School of Medicine, The University of Tokyo, Bunkyo-ku, Tokyo 113-0033, Japan; hiroco007@m.u-tokyo.ac.jp (H.N.); prisch@m.u-tokyo.ac.jp (P.C.); thaya@m.u-tokyo.ac.jp (T.H.); 2 Department of Microbiology, Faculty of Medicine, Shimane University, Izumo 693-8501, Japan; iizasah@med.shimane-u.ac.jp (H.I.); yosiyama@med.shimane-u.ac.jp (H.Y.); 3 Laboratory of Virology, Institute of Microbial Chemistry, Microbial Chemistry Research Foundation, Shinagawa-ku, Tokyo 141-0021, Japan; 4 Center for Infectious Cancers, Institute for Genetic Medicine, Hokkaido University, Kita-ku, Sapporo 060-0815, Japan

**Keywords:** PAR1b, multimerization, nucleic acids, kinase activation, EBER, gastric cancer

## Abstract

PAR1b is a cytoplasmic serine/threonine kinase that controls cell polarity and cell–cell interaction by regulating microtubule stability while mediating cytoplasmic-to-nuclear translocation of BRCA1. PAR1b is also a cellular target of the CagA protein of *Helicobacter pylori,* which leads to chronic infection causatively associated with the development of gastric cancer. The CagA-PAR1b interaction inactivates the kinase activity of PAR1b and thereby dampens PAR1b-mediated BRCA1 phosphorylation, which reduces the level of nuclear BRCA1 and thereby leads to BRCAness and BRCAness-associated genome instability underlying gastric carcinogenesis. While PAR1b can multimerize within the cells, little is known about the mechanism and functional role of PAR1b multimerization. We found in the present study that PAR1b was multimerized in vitro by binding with nucleic acids (both single- and double-stranded DNA/RNA) via the spacer region in a manner independent of nucleic-acid sequences, which markedly potentiated the kinase activity of PAR1b. Consistent with these in vitro observations, cytoplasmic introduction of double-stranded DNA or expression of single-stranded RNA increased the PAR1b kinase activity in the cells. These findings indicate that the cytoplasmic DNA/RNA contribute to nuclear accumulation of BRCA1 by constitutively activating/potentiating cytoplasmic PAR1b kinase activity, which is subverted in gastric epithelial cells upon delivery of *H. pylori* CagA oncoprotein.

## 1. Introduction

Partitioning-defective 1 (PAR1), a family of cytoplasmic serine/threonine kinases evolutionally conserved from yeast to mammalian cells, plays a diverse role in cellular processes such as cell polarity, microtubule dynamics, cell cycle control, cell metabolism, and genome stability [1,2]. Mammalian PAR1 was originally discovered as microtubule affinity-regulating kinase (MARK), that is comprised of four homologous members, PAR1c/MARK1, PAR1b/MARK2, PAR1a/MARK3, and PAR1d/MARK4. Of these, PAR1b/MARK2 is predominantly expressed in many cell types, including epithelial cells. In the polarized alimentary epithelia, PAR1b/MARK2 is localized to the basolateral plasma membrane of epithelial cells to establish and maintain apico–basal polarity [3,4]. In neuronal cells, PAR1/MARK phosphorylates the microtubule-associated protein (MAP) tau at the KXGS motifs (S262) in the C-terminal repeat region [5,6], which causes detachment of tau from microtubules and subsequent microtubule destabilization. Importantly, hyperphosphorylation of tau has been associated with neurodegenerative diseases collectively known as tauopathies, especially Alzheimer’s disease [7].

The kinase catalytic domain of the PAR1 family members is located in the N-terminus, which is followed by the accessory UBA domain, a large (>300 aa) flexible linker called the spacer region, and the C-terminal kinase-associated 1 (KA1) domain [1]. Multiple mechanisms exist in regulating the kinase activity of PAR1b. Phosphorylation at T208 in the activation loop enhances the basal kinase activity [8,9] while phosphorylation at T212, another site in the activation loop, is inhibitory [10]. PAK5 inhibits the PAR1b kinase activity by binding to its catalytic domain [11]. Similarly, the *Helicobacter pylori* CagA oncoprotein directly binds to and thereby inhibits the kinase catalytic domain of the PAR1 family, especially PAR1b [12,13]. Another layer of regulation is provided by autoinhibition. The kinase-associated 1 (KA1) domain of PAR1 kinases autoinhibits the catalytic domain via intramolecular association [14,15]. This autoinhibition can be relieved by the association of KA1 domain with phospholipids. In addition, autoinhibitory effects of the spacer region on the catalytic domain can be relieved by the binding of DAPK to the spacer region of PAR1b [16].

We recently found that *H. pylori* CagA-mediated inhibition of the kinase activity of PAR1b through complex formation prevents cytoplasmic-to-nuclear translocation of the BRCA1 tumor suppressor by inhibiting BRCA1 phosphorylation on S616. This inhibition resulted in the loss of nuclear BRCA1 and subsequent destabilization of DNA replication fork, which causes fork arrest and collapse that leads to DNA-double strand breaks (DSBs) [2]. CagA-induced DSBs are repaired by error-prone non-homologous mechanisms due to the absence of BRCA1 in the nucleus. The cellular state, termed BRCAness, leads to hyperaccumulation of genome mutations, which underlies development of gastric cancer upon infection with *cagA*-positive *H. pylori* [2].

The above-described findings indicate that PAR1b kinase activity is critically associated with genome stability. In the present study, we unexpectedly found that nucleic acids, DNA and RNA [either single-stranded (ss) or double-stranded (ds)], mediate the multimerization and catalytic activation of PAR1b by binding to its spacer region independently of their nucleotide sequences. The finding adds PAR1b to a very short list of kinases, in which kinase activities are regulated by nucleic acids such as the dsRNA-dependent PKR [17] and the dsDNA-dependent DNA-PK [18]. As perturbation of the PAR1b kinase activity has been shown to be critically involved in the pathogenesis of human diseases, including cancers [13,19,20] and neurodegenerative diseases [7], elucidation of a hitherto-unidentified mechanisms underlying kinase activation of PAR1b may benefit the development of innovative therapeutics against these intractable human disorders.

## 2. Results

### 2.1. Nucleic Acids Mediate Multimerization of PAR1b

**Figure 1:**
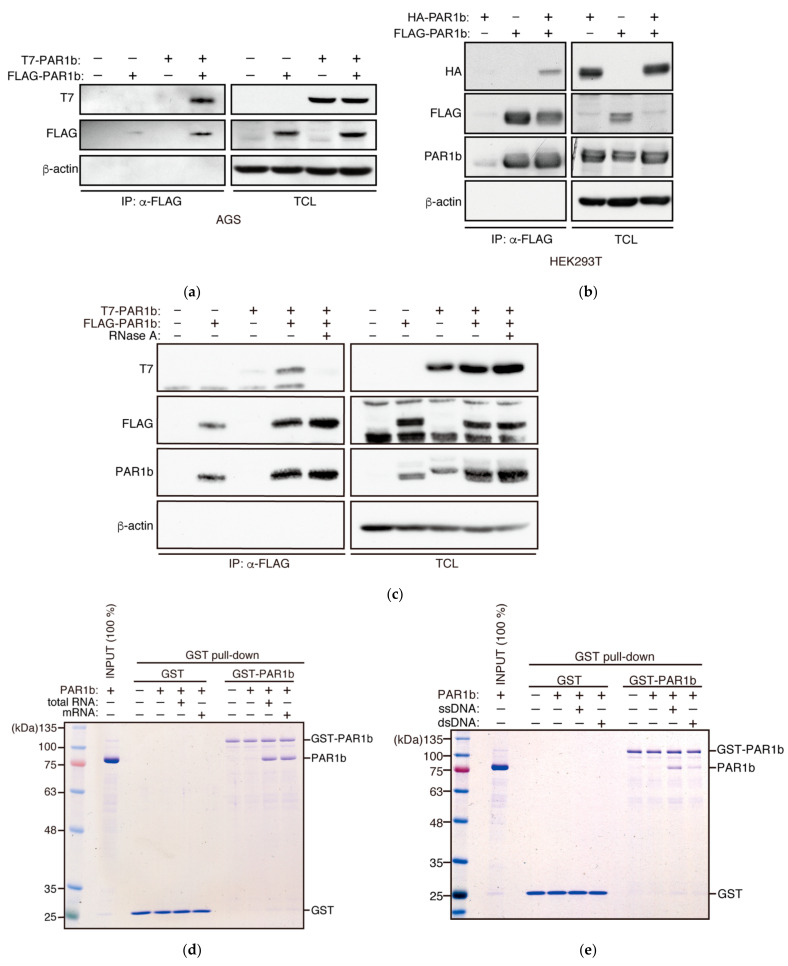
Nucleic acids mediate multimerization of PAR1b. (**a**) Multimerization of PAR1b in AGS cells. AGS cells were transiently transfected with expression vectors for FLAG-tagged PAR1b and T7-tagged PAR1b. After 24 h, total cell lysates (TCL) were prepared and subjected to immunoprecipitation using the anti-FLAG antibody. Immunoprecipitates (IP) were analyzed by immunoblotting and detected by the indicated antibodies. (**b**) Multimerization of PAR1b in HEK293T cells. Immunoprecipitation was performed with HEK293T cells transiently transfected with expression vectors for FLAG-tagged PAR1b and HA-tagged PAR1b. (**c**) Multimerization of PAR1b in AGS cells is mediated by RNA. TCL was treated with RNase A prior to immunoprecipitation. (**d**) Reconstitution of RNA-mediated multimerization of PAR1b. GST pull-down was performed by mixing GST-PAR1b bound Glutathione Sepharose beads with 100 nM PAR1b in the presence of 1 µg/mL total RNA or mRNA purified from AGS cells. A sample containing only the GST-PAR1b bound beads was analyzed as the input control for GST-PAR1b. (**e**) DNA can also mediate multimerization of PAR1b. GST pull-down between GST-PAR1b and 100 nM PAR1b was performed in the presence of 1 µg/mL ssDNA or dsDNA prepared from AGS cells. A sample containing only the GST-PAR1b bound beads was analyzed as the input control for GST-PAR1b.

We previously showed that PAR1b can exist in a multimerized form in human gastric epithelial cells, which potentiates oncogenic potential of *Helicobacter pylori* CagA by stabilizing its binding with the prooncogenic tyrosine phosphatase SHP2 [13,21]. We verified PAR1b multimerization by transiently co-expressing FLAG epitope-tagged PAR1b and T7 epitope-tagged PAR1b in AGS human gastric epithelial cells. As previously reported, immunoprecipitation of the cell lysates with FLAG-PAR1b co-precipitated T7-PAR1b, showing the presence of PAR1b homomultimers in AGS cells (Figure 1a). Formation of PAR1b multimers was also observed in HEK293T cells (Figure 1b), suggesting that PAR1b multimerization is not a unique feature to specific cell types.

Previous reports have shown that the N-terminal fragment of PAR1b, containing the catalytic and UBA domains, was monomeric in solution [19,22]. The PAR1b protein ectopically expressed in AGS cells for the co-immunoprecipitation assay shown in Figure 1 was full-length. Accordingly, we next investigated whether full-length PAR1b could multimerize in solution. To this end, we purified recombinant full-length PAR1b from *E. coli* and examined whether PAR1b spontaneously multimerizes in solution by a pull-down assay using GST-fused full-length PAR1b (Figure S1). The result of the experiment revealed that there was no physical interaction between full-length PAR1b and GST-PAR1b in in vitro solution, suggesting that PAR1b requires either a posttranslational modification or another cellular component(s) to undergo multimerization in the cells.

During the course of optimizing conditions for purifying recombinant PAR1b from *E. coli*, we noticed that PAR1b bound non-specifically to *E. coli* RNA under physiological salt conditions, resulting in the aggregation and precipitation of recombinant PAR1b that made its purification rather difficult. Raising the ionic strength of the lysis buffer released PAR1b from RNA and solved this purification problem. Since a number of nucleic acid-binding proteins are known to bind non-specifically to nucleic acids in *E. coli*, we asked if RNA is involved in PAR1b multimerization in AGS cells. Strikingly, treating the total cell lysate with Rnase A prior to co-immunoprecipitation markedly impaired PAR1b multimerization (Figure 1c). The finding indicated that binding of RNA to PAR1b is required for PAR1b multimerization in mammalian cells.

To investigate what kind of RNA species can mediate the formation of PAR1b multimers, we mixed recombinant GST-PAR1b and PAR1b in the presence of either total RNA or mRNA that was purified from AGS cells, followed by a GST pull-down assay (Figure 1d). As a result, both RNA species were capable of reconstituting PAR1b multimerization in vitro, indicating that the ability of RNA to induce PAR1b multimerization was independent of RNA species or particular RNA sequences. The result also suggested that posttranslational modification of PAR1b in mammalian cells is not required for the formation of PAR1b multimers.

The fact that PAR1b multimerization is mediated by RNA in a non-sequence specific manner reminded us of how polyanions such as RNA, heparin, and heparan sulphate can promote the aggregation of tau, a substrate of PAR1b, via electrostatic interactions in vitro [23,24]. Thus, we sought to test whether polyanions other than RNA could also mediate PAR1b multimerization. Since heparin and heparan sulphate are constituents of the extracellular matrix, we tested DNA, another polyanion that could exist in the cytoplasm where PAR1b is localized. Recent studies have suggested that DNA fragments, often as a consequence of micronuclei rupture or DNA damage, can exist in the cytoplasm of aging cells [25]. We performed a GST pull-down assay in the presence of either ssDNA or dsDNA and found that both species of DNA could facilitate multimerization, although ssDNA promoted multimerization more than dsDNA (Figure 1e). One explanation for this difference may be that we added the same mass concentration of nucleic acids into the mixture. This means that in terms of molarity, only half the number of polyanion molecules exist for dsDNA compared to ssDNA. Moreover, total RNA and ssDNA promoted the multimerization of PAR1b to similar degrees, which suggested that the number of polyanion molecules is important for PAR1b multimerization, not its chemical makeup (Figure S2). Overall, these findings indicated that RNA and DNA, most likely as polyanions, mediate PAR1b multimerization at least in the context of the GST pull-down assay.

### 2.2. PAR1b Multimerizes through the Spacer Region

**Figure 2:**
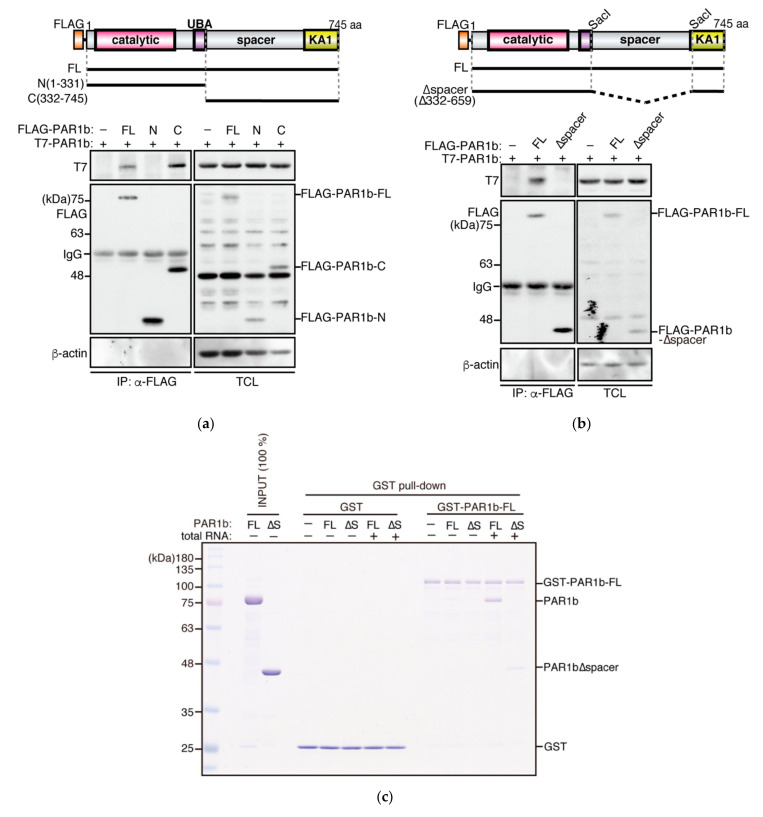
Mapping the multimerization domain of PAR1b. (**a**) The C-terminus of PAR1b is required for multimerization in cells. Schematic diagram of full-length (FL) FLAG-PAR1b and its N-terminal and C-terminal truncations *(top)*. Amino acid residue numbers refer to the 745 aa isoform of PAR1b (see Methods). AGS cells transiently transfected with expression vectors for the FLAG-PAR1b mutants and T7-PAR1b were subjected to immunoprecipitation *(bottom)*. (**b**) The spacer region is required for multimerization in cells. Schematic diagram of FLAG-PAR1b-∆spacer *(top)*. AGS cells transiently transfected with the expression vectors for FLAG-PAR1b-∆spacer and T7-PAR1b were subjected to immunoprecipitation *(bottom)*. (**c**) PARb multimerizes primarily through the spacer region in the presence of nucleic acid. GST-PAR1b-FL bound Glutathione Sepharose beads were mixed with 100 nM PAR1b-FL or PAR1b-∆spacer in the presence of 1 µg/mL total RNA and a GST pull-down assay was performed.

To determine which region of PAR1b is required for multimerization, we constructed FLAG expression vectors to express the N-terminal fragment of PAR1b [PAR1b-N(1-331)], which consists of the catalytic and the UBA domains, or the C-terminal fragment of PAR1b [PAR1b-C(332-745)], which is comprised of a large region of unknown function called the spacer region and the highly conserved kinase-associated 1 (KA1) domain (Figure 2a). Co-expression of these mutant proteins with full-length PAR1b (PAR1b-FL) in AGS cells, followed by immunoprecipitation, showed that the C-terminal fragment is essential for PAR1b multimers to form.

The spacer region is predicted to be highly basic (pI 10.8) and intrinsically disordered [1,26] (Figure S3). As such regions, including that of tau, have been identified as nucleic acid-binding domains in recent years [27,28], it prompted us to examine whether the spacer region mediates PAR1b multimerization. We designed an expression vector for PAR1b-∆spacer, which lacks the spacer region, based on two previous studies which defined the regions for UBA and KA1 domains [22,29] (Figure 2b). A co-immunoprecipitation assay using AGS cells that transiently expressed PAR1b-∆spacer showed that PAR1b-∆spacer lost its ability to interact with PAR1b-FL, indicating that the spacer region is essential for the formation of PAR1b multimers in cells. Furthermore, to investigate whether there is a specific sequence in the spacer region that is responsible for the multimerization, we made a series of constructs to express PAR1b that contained partially deleted forms of the spacer region and performed a co-immunoprecipitation assay (Figure S4). All three forms of PAR1b with partial deletions of the spacer region were able to multimerize, indicating that a specific sequence is not attributable to PAR1b multimerization. This result further supported the idea that the interaction between PAR1b and nucleic acids is driven electrostatically by the positively charged spacer region of PAR1b and the negatively charged polyanions, analogous to its substrate tau.

To confirm whether the spacer region mediated PAR1b multimerization by direct interaction with nucleic acids, we performed a GST pull-down assay using recombinant PAR1b-∆spacer protein. As Figure 2c shows, the formation of multimers was diminished when PAR1b-∆spacer was mixed with PAR1b-FL in the presence of total RNA. Together with the findings above, the PAR1b spacer region is the multimerization domain of PAR1b which mediates multimerization through its direct interaction with nucleic acids.

### 2.3. Nucleic Acids Potentiate the Kinase Activity of PAR1b through the Spacer Region

**Figure 3:**
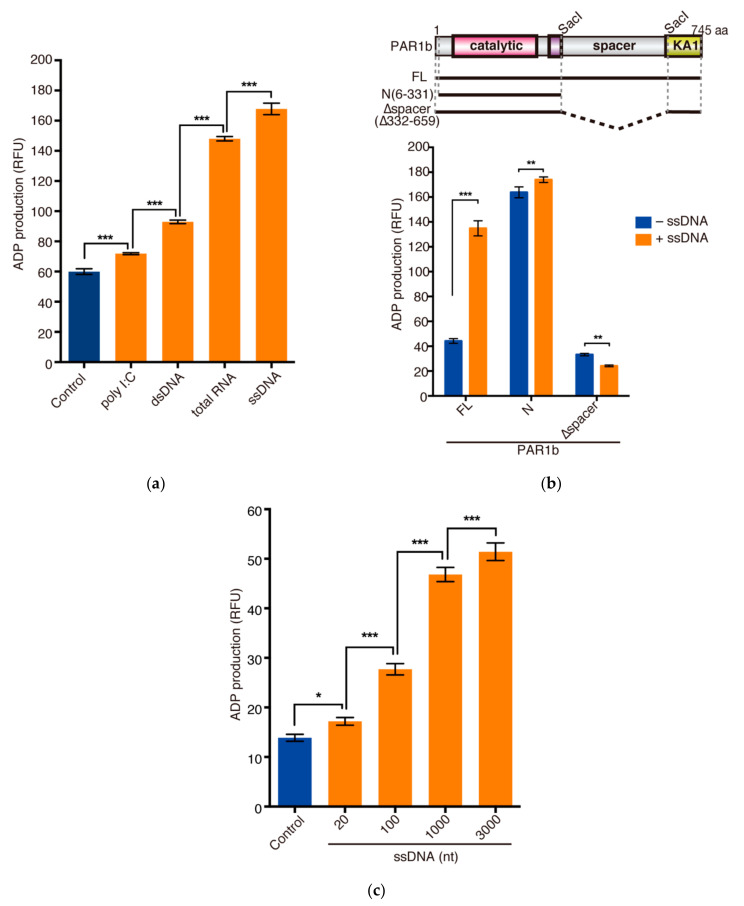
Nucleic acids enhance PAR1b kinase activity. (**a**) The effect of various nucleic acids on the kinase activity of recombinant PAR1b in an in vitro kinase assay. 200 nM PAR1b-FL was subjected to an in vitro kinase assay in the presence of 40 ng/µL nucleic acids as indicated. Total RNA, ssDNA, and dsDNA were purified from AGS cells. (**b**) The spacer region is required for the nucleic acid-dependent potentiation of PAR1b kinase activity. 200 nM of the mutant PAR1b proteins indicated (*top*) were subjected to an in vitro kinase assay in the presence or absence of 40 ng/µL ssDNA from AGS cells (*bottom*). (**c**) PAR1b kinase activity is dependent on the length of nucleic acids. 50 nM of PAR1b-FL was subjected to an in vitro kinase assay in the presence of 10 ng/µL ssDNA of different lengths as indicated. Kinase activities were quantified through the amount of ADP produced, which was measured using a fluorescence spectrometer (relative fluorescent units, RFU). Error bars represent mean ± SD, *n* = 4. * *p* < 0.05, ** *p* < 0.01, *** *p* < 0.001, one-way ANOVA, post-hoc Tukey’s test (**a**,**c**) or two-way ANOVA, post-hoc Sidak’s test (**b**).

We next investigated whether nucleic acids, which promote PAR1b multimerization, could potentiate the kinase activity of PAR1b. Using the tau repeat 1 (TR1) peptide as a substrate, we performed an in vitro kinase assay to compare the kinase activities of PAR1b in the presence of ssDNA, dsDNA, total RNA, and poly I:C, a synthetic analog of dsRNA. Intriguingly, the single-stranded nucleic acids, ssDNA and total RNA, markedly potentiated the kinase activity of PAR1b (2.8-fold and 2.5-fold, respectively) (Figure 3a). Double-stranded nucleic acids, dsDNA and poly I:C, had modest effects on the kinase activity (1.6-fold and 1.2-fold, respectively).

To determine whether the enhancement of kinase activity could be ascribed to the spacer region of PAR1b, we performed an in vitro kinase assay using recombinant PAR1b-N(6-331) and PAR1b-∆spacer in the presence of ssDNA. The flexibility of the spacer region is thought to allow the KA1 domain to associate with the catalytic domain of PAR1b *in cis* to autoinhibit the kinase activity of PAR1 orthologs [14,15]. As expected, without the autoinhibitory effect of KA1, PAR1b-N(6-331) was constitutively active, and the addition of ssDNA only had a minor effect on its kinase activity (Figure 3b). Furthermore, we also observed low kinase activity for PAR1b-∆spacer that was comparable to the basal activity of PAR1b-FL. As the spacer region was completely deleted in PAR1b-∆spacer, we speculate that, without the flexibility of the spacer region, the KA1 domain acts *in trans* to inhibit the kinase activity of another PAR1b-∆spacer molecule. Interestingly, the addition of nucleic acids could not enhance the activity of PAR1b-∆spacer. This indicated that the spacer region is essential for the potentiation of kinase activity by nucleic acids. Binding of nucleic acids to the spacer region may release the KA1 domain from the catalytic domain to potentiate the kinase activity of PAR1b-FL.

The function of some RNA and DNA sensors is dependent on the length of their ligands [30,31,32]. Therefore, we tested whether the length of nucleic acids also affects the kinase activity of PAR1b. We prepared linear ssDNA of different lengths (20, 100, 1000, 3000 nt) and performed an in vitro kinase assay (Figure 3c). A total of 20 nt and 100 nt fragments induced a modest potentiation of PAR1b kinase activity (1.2-fold and 2.0-fold, respectively), while longer fragments 1000 nt and 3000 nt gave the strongest effects (3.4-fold and 3.7-fold, respectively). This experiment demonstrated that the highest PAR1b kinase activities are achieved with longer nucleic acids in the in vitro kinase assay.

### 2.4. PAR1b Can Multimerize in a DNA-Dependent Manner in Cells

**Figure 4:**
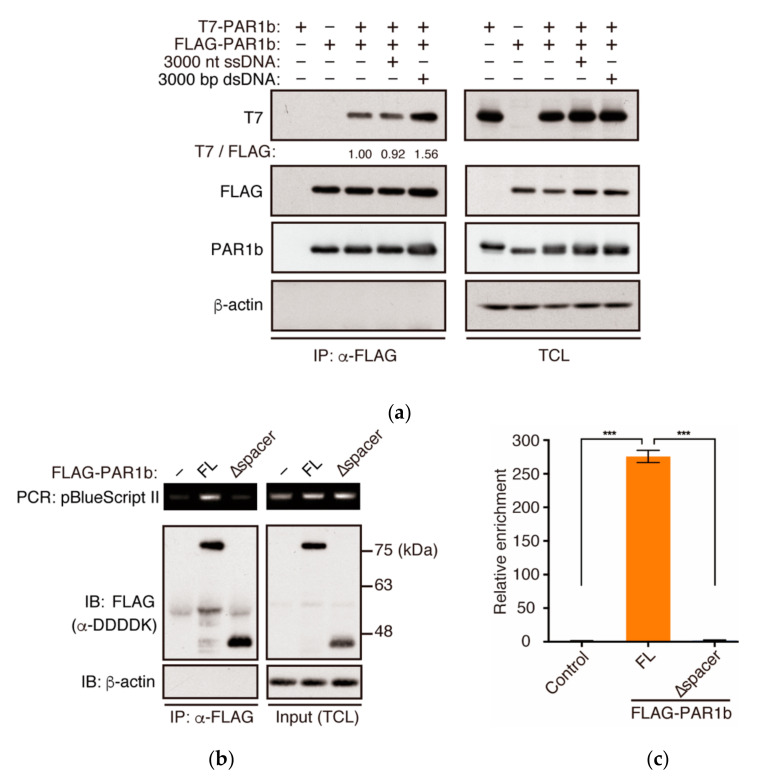
PAR1b can multimerize in a nucleic acid-dependent manner in the cell. (**a**) dsDNA promotes multimerization of PAR1b in cells. AGS cells were transiently transfected with expression vectors for T7-PAR1b and FLAG-PAR1b. After 24 h, cells were transfected with 2 µg/mL 3000 nt ssDNA or 3000 bp dsDNA for 5 h followed by immunoprecipitation. (**b**) PAR1b can interact with DNA directly through the spacer region in cells. AGS cells were transiently transfected with FLAG-PAR1b expression vectors. After 24 h, cells were transfected with a mixture of pBlueScript II and salmon sperm dsDNA at a ratio of 1:1000 for 2 h before harvest and subsequent DNA-immunoprecipitation. pBlueScript II was detected by PCR using specific primers. (**c**) Quantitative PCR analysis of DNA immunoprecipitants from (**b**). Error bars represent mean ± SD, *n* = 3. *** *p* < 0.001, one-way ANOVA, post-hoc Tukey’s test.

Recently, DNA has been shown to accumulate in the cytoplasm of cells with DNA damage, senescent cells, and cancer cells [25,33]. Therefore, we asked whether the introduction of DNA to the cytoplasm can promote the multimerization of PAR1b in cells. To this end, we transiently expressed FLAG-PAR1b and T7-PAR1b in AGS cells and then transfected the cells with an equal mass of either ssDNA of 3000 nt in length or dsDNA of 3000 bp in length (Figure 4a). We were surprised to find that contrary to the results using recombinant proteins, where ssDNA was more potent in inducing PAR1b multimerization and kinase activity than dsDNA, PAR1b multimerization was promoted by dsDNA, and not ssDNA, in the cellular context. One explanation for this may be because ssDNA, compared to dsDNA, is more unstable and prone to nuclease attack in the cytoplasm. Previous studies have shown that there are cell intrinsic mechanisms in which ssDNA is digested by TREX1 exonuclease in the cytoplasm and excess ssDNA can be sequestered into the nucleus by RPA and Rad51 [34,35]. Such mechanisms may prevent ssDNA from promoting PAR1b multimerization inside the cell.

Introduction of foreign DNA into the cell cytoplasm may trigger innate immune responses that indirectly affect PAR1b multimerization. To ascertain that DNA bound directly to PAR1b to promote multimerization inside the cell, we transfected a small amount of plasmid DNA, mixed with carrier DNA at a ratio of 1:1000, and examined whether it could co-immunoprecipitate with PAR1b. Indeed, the plasmid DNA specifically co-immunoprecipitated with PAR1b as detected by conventional PCR and quantitative PCR using specific primers (Figure 4b,c). Furthermore, this interaction was dependent on the spacer region, as PAR1b-∆spacer could not interact with the plasmid. Hence, these results suggested that DNA directly induced the multimerization of PAR1b.

### 2.5. dsDNA Potentiates the Kinase Activity of PAR1b

**Figure 5:**
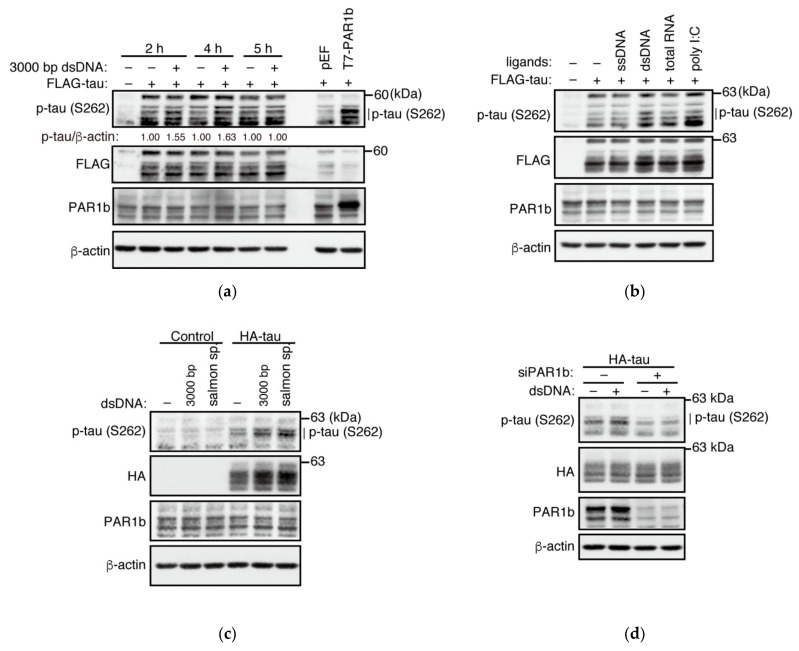
Nucleic acids can enhance the kinase activity of PAR1b in the cell. (**a**) Transfection of dsDNA induces phosphorylation of tau at S262 in cells in a time-dependent manner. AGS cells were transiently transfected with an expression vector for FLAG-tau. After 24 h, cells were transfected with 2 µg/mL linearized 3000 bp dsDNA for 2, 4 and 5 h before cell lysis. Cells co-transfected with expression vectors for FLAG-tau and T7-PAR1b are used as positive control (*final lane*). Phosphorylation of tau was quantified with an image analyzer and normalized to its control at each time point. (**b**) The effect of various nucleic acid ligands on the phosphorylation of tau. AGS cells were transiently transfected with an expression vector for FLAG-tau followed by transfection with 2 µg/mL of ssDNA from salmon sperm, dsDNA from salmon sperm, total RNA from AGS cells or poly I:C for 2 h. (**c**) dsDNA induces phosphorylation of tau in AGS cells stably expressing HA-tau. The stable cell line was transfected with 2 µg/mL linearized 3000 bp dsDNA or dsDNA from salmon sperm. (**d**) Phosphorylation of tau at S262 is dependent on PAR1b. AGS cells stably expressing HA-tau were treated with PAR1b-specific siRNA for 48 h and then transfected with 2 µg/mL dsDNA from salmon sperm for 2 h.

Since dsDNA was able to promote the multimerization of PAR1b, we next examined whether it could potentiate PAR1b kinase activity inside the cell. We transiently expressed FLAG-tagged tau protein in AGS cells and transfected 3000 bp linearized dsDNA for 2, 4, and 5 h. Phosphorylation levels of tau at S262 were elevated at earlier timepoints 2 h and 4 h, while the change was lost by 5 h (Figure 5a). Additionally, consistent with previous reports, phosphorylation of S262 stabilized the level of total tau [36]. This suggested that the activity of PAR1b responds quickly to elevated levels of dsDNA in the cytoplasm.

We also investigated whether other nucleic acids could potentiate the phosphorylation of tau at S262. Consistent with the previous co-immunoprecipitation experiment, transfection of dsDNA could promote the phosphorylation of tau at S262 better than ssDNA (Figure 5b). Furthermore, poly I:C was also able to induce phosphorylation of tau while the total RNA induced a modest effect. Heightened response to double-stranded nucleic acids may reflect the stability of these species in the cell as already mentioned above.

One reservation that we had from transient expression of tau was that the expression vector for tau, itself a dsDNA, could have an impact on the activity of PAR1b. Hence, to avoid introducing expression vectors prior to the transfection of nucleic acid ligands, we constructed stable cell lines expressing HA-tau and tested whether dsDNA could potentiate PAR1b activity (Figure 5c and Figure S5). Transfection of two types of dsDNA, linearized plasmid or sheared salmon sperm, increased the level of phosphorylated tau at S262, which supported the previous results from the transient expression of tau. Furthermore, knockdown of PAR1b resulted in the reduction in phosphorylated tau in the presence of dsDNA, indicating that PAR1b is essential for the increase in phosphorylation of tau at S262 (Figure 5d).

### 2.6. The Effect of Epstein–Barr Virus EBERs on PAR1b

**Figure 6:**
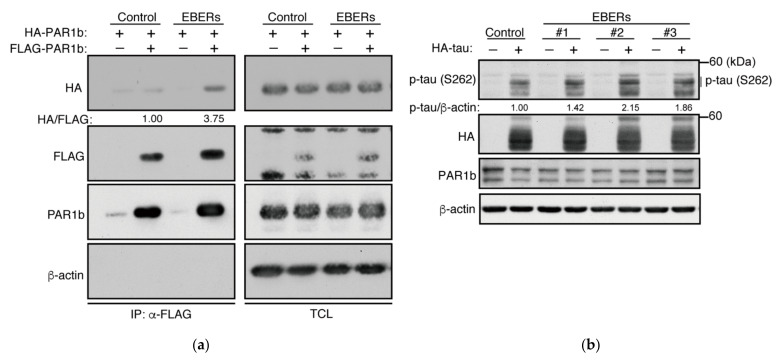
EBERs from Epstein–Barr virus can promote PAR1b multimerization and enhance its kinase activity. (**a**) Multimerization of PAR1b in AGS cells stably expressing EBERs. AGS cells stably expressing EBERs were transiently transfected with expression vectors for FLAG-tagged PAR1b and HA-tagged PAR1b and immunoprecipitation was performed. (**b**) Expression of EBERs can potentiate the phosphorylation of tau at S262. HA-tau expression vectors were transiently transfected to three independent clones of AGS cells stably expressing EBERs.

The Epstein–Barr virus (EBV) accounts for 9% of all gastric carcinomas and has recently been shown to form a distinct molecular subtype of gastric cancer [37,38]. Epstein–Barr virus-associated gastric cancer (EBVaGC), in which the virus is in its latency phase, is characterized by the high expression of non-coding RNAs. Among these, EBV-encoded small RNAs (EBERs), EBER1 and EBER2, which are 167 and 173 nucleotides in length, respectively, are the most abundant transcripts expressed (~10^7^ molecules/cell) [39,40]. While EBER1 and EBER2 share only 54% similarity in their primary sequence, they are both extensively double-stranded with a number of short stem-loop structures. EBERs are known to be expressed primarily in the nucleus, yet they have been reported to interact with a number of cytoplasmic proteins such as La protein, PKR, and RIG-I [40,41,42]. Therefore, we wondered whether abundantly expressed viral transcripts such as EBERs could mediate multimerization of PAR1b inside the cell. To this end, we constructed AGS cell lines that stably express both EBERs. Expression of HA-PAR1b and FLAG-PAR1b in a cell line stably expressing the EBERs showed that the multimerization of PAR1b was increased in the EBER-expressing cells compared to the control (Figure 6a). Furthermore, we confirmed that EBERs can enhance the activity of PAR1b in these cells (Figure 6b). Thus, abundantly expressed viral transcripts such as EBERs can mediate multimerization of PAR1b and enhance its kinase activity.

## 3. Discussion

In the present study, we have shown that the catalytic activity of PAR1b is potentiated by its multimerization, which is mediated via nucleic acids. Whereas other nucleic acid-dependent kinases such as PKR and DNA-PK have specific nucleic acid-binding motifs [43,44], PAR1b is unique in that its binding to nucleic acids requires a large, basic, and intrinsically disordered region termed the spacer region. The finding is consistent with recent reports showing that nearly half of the RNA-binding domains of proteins are mapped to intrinsically disordered regions [45] and that up to 70% of DNA-binding proteins harbor intrinsically disordered tails [46], in which basic residues play key roles in the protein–nucleic acid interactions. Comprehensive analysis using mass spectrometry has identified PAR1b as a potential RNA-binding protein and indicated the PAR1b spacer region as the candidate RNA-binding region [27,45], strongly supporting our finding that PAR1b is a novel kinase, the spacer region of which binds to nucleic acids. 

The kinase activity of PAR1b is regulated through its intramolecular interaction between the N-terminal catalytic domain and the C-terminal KA1 domain [14,15]. Since the spacer region potentiates the kinase activity of PAR1b in the presence of nucleic acids, we speculate that the electrostatic interaction between the positively charged spacer region and negatively charged nucleic acids circumvent the intramolecular autoinhibitory interaction between the catalytic domain and the KA1 domain. Our findings therefore propose a new mode of PAR1b kinase regulation. It remains to be determined whether other members of the PAR1 family share the same regulatory mechanism. Interestingly, tau is also a basic protein and interacts with RNA through electrostatic interactions [24,47]. This notion indicates that RNA acts as a scaffold that brings the activated PAR1b kinase and its substrate MAPs together for efficient phosphorylation. Our results also showed that the activity of PAR1b is dependent on the length of nucleic acids. Although we do not know the underlying mechanism, many cytosolic nucleic acid sensors have been shown to oligomerize on nucleic acids in a cooperative manner, such as the dsDNA sensors cGAS [48] and IFI16 [31], and the dsRNA sensor Mda5 [32]. Notably, cGAS has been shown to enhance its enzymatic activity in a DNA length-dependent manner [30,48]. Cooperative oligomerization on nucleic acids, coupled with the enhanced enzymatic activity, may be an attractive mechanism for the potentiation of PAR1b depending on the length of nucleic acids.

The results of the present work showed that RNA mediates the multimerization of PAR1b in the cell under physiological conditions. This is presumably due to the fact that in the physiological state, RNA is the primary nucleic acid present in the cell cytoplasm where PAR1b is localized. PAR1b–RNA interactions may keep the kinase activity of PAR1b above a certain threshold level so that PAR1b is capable of regulating microtubule dynamics [5], cell polarity [3,4], and chromosome maintenance [2] in the steady state conditions. Specifically, constitutively activated PAR1b is required for the cytoplasmic-to-nuclear translocation of BRCA1, where it stabilizes DNA replication forks to prevent endogenous DSB induction while it mediates error-free homologous recombination-mediated DSB repair.

In addition to RNA, DNA can also activate the kinase activity of PAR1b in the in vitro kinase assay and in the cell. The finding may have important implications as the presence of DNA in the cytoplasm poses potential dangers to the cell. Cell-intrinsic mechanisms exist to sense foreign and endogenous DNAs in the cytoplasm, such as the cGAS-STING pathway and the pathogen-associated molecular patterns (PAMPs). While foreign DNA is associated with viral infections, endogenous cytoplasmic DNA typically includes micronuclei and chromatin fragments, which are caused by chromosomal instability and malfunctioning of the nuclear membrane, respectively [25]. Their presence leads to the aberrant activation of the innate immunity and has been linked to chronic age-related diseases such as cancer and neurodegenerative diseases. As we have found that dsDNA can multimerize and potentiate the kinase activity of PAR1b, we postulate that PAR1b is upregulated in response to cellular emergencies in which DNA is leaked to the cytoplasm. Future studies should focus on the downstream effectors of PAR1b to identify its exact role in the presence of abnormal nucleic acids in the cytoplasm. Whether this is a cellular defense mechanism or an aberrant activation that leads to pathologic consequences warrants further investigation.

A comprehensive molecular characterization of gastric cancer has been reported by the TCGA Research Network [38]. The study created four subtypes for the molecular classification of gastric cancer: tumors positive for Epstein–Barr virus (EBV) (EBV-positive; 9%), microsatellite instable tumors (MSI; 22%), genomically-stable tumors (GS; 20% samples), and tumors with chromosomal instability (CIN; 50%). Of these, CIN type and GS type roughly correspond to intestinal type and diffuse type, respectively, by Lauren’s classification. Notably, CIN type is genetically characterized by the mutation of *TP53* (71%), which indicates the critical role of *H. pylori* CagA/PAR1b-mediated BRCAness in the development of CIN-type gastric cancer. In contrast, EBV-positive gastric cancer rarely exhibits *TP53* mutation, indicating little contribution of CagA-mediated BRCAness in its development. The notion is also consistent with the finding that BRCA mutation signature is almost specifically observed in CIN type (intestinal type) gastric cancer. In EBV-positive gastric cancer, deregulated activation of PAR1b by EBV-encoded RNAs such as EBERs may underscore neoplastic transformation of cells as opposed to the case of intestinal-type (CIN type) gastric cancer in which *H. pylori* CagA-induced inactivation of PAR1b and subsequent induction of BRCAness is critically involved in the development of cancer. Hyperactivation of PAR1 family kinases is also observed in several cancer types; PAR1d is upregulated in glioblastoma, hepatocarcinoma, breast cancer, and lung cancer cells [49,50,51]. PAR1b is often overexpressed in non-small cell lung carcinoma [20]. Accordingly, either excess or impaired kinase activity of PAR1 may contribute to tumor progression. A critical question is then how deregulated activation of PAR1 kinase activity predisposes various cell types, including gastric epithelial cells, to neoplastic transformation.

## 4. Materials and Methods

### 4.1. Bacterial Expression Vectors

pGEX-6P-2 and pGEX-6P-3 (GE Healthcare, Chicago, IL, USA) expression vectors for GST-PAR1b-FL and GST-PAR1b-N(6-331) (equivalent to PAR1b(39-364) of the longest isoform), respectively, have been described previously [19]. The GST-PAR1b-∆spacer expression vector was made by excising the SacI-SacI fragment from the GST-PAR1b-FL expression vector. An expression vector for HA-PAR1b-FL was made by cloning in a sequence for HA tag, generated by PCR, into pSP65SRα-FLAG-PAR1b-FL to replace the N-terminal FLAG sequence. It should be noted that all bacterial constructs used in this study contain a C-terminal hexahistidine sequence for purification purposes.

### 4.2. Antibodies

Anti-FLAG monoclonal antibody (M2, Sigma-Aldrich, St. Louis, MO, USA), anti-DDDDK polyclonal antibody (MBL, Woburn, MA, USA), anti-HA monoclonal antibodies (6E2 or C29F4, Cell Signaling Technology, Danvers, MA, USA), omni-probe (T7) polyclonal antibody (M-21, Santa Cruz Biotechnology, Dallas, TX, USA), anti-MARK2 monoclonal antibody (EPR8553, Abcam, Cambridge, UK), anti-beta actin polyclonal antibody (C-11, Santa Cruz Biotechnology, Dallas, TX, USA), anti-beta actin monoclonal antibody (8H10D10, Cell Signaling Technology, Danvers, MA, USA), and anti-phosphorylated tau S262 monoclonal antibody (TIP1-35, Fujifilm Wako, Osaka, Japan) were used as primary antibodies for immunoprecipitation and immunoblotting.

### 4.3. Protein Expression and Purification

Protein expression in *E. coli* BL21 and subsequent purification of PAR1b-N were performed as previously described [19]. PAR1b-∆spacer was expressed and purified using the same protocol. PAR1b-FL was expressed and purified as previously described [19], with some modifications to ensure RNA-free preparations without the use of nucleases that may interfere with downstream applications. For the initial lysis, a high salt lysis buffer (PBS, 2% (*v*/*v*) Tween 20, 1% (*v*/*v*) Triton X-100, 1 mM EDTA, 1 mM DTT, 850 mM NaCl) + 0.3 mg/mL Benzamidine was used. Cleared lysate was mixed with Ni Sepharose excel beads (GE Healthcare, Chicago, IL, USA) and washed thoroughly with wash buffer (high salt lysis buffer + 20 mM imidazole), followed by elution (high salt lysis buffer + 500 mM imidazole). After adjusting the salt concentration to 500 mM NaCl, the subsequent GST purification steps were performed. The final incubation with 28 U/mL PreScission Protease (GE Healthcare, Chicago, IL, USA) was performed in 50 mM Tris-Cl, pH7.5, 500 mM NaCl, 1 mM EGTA, 1 mM DTT at 4 °C overnight.

### 4.4. GST Pull-Down Assay

GST-PAR1b-FL was bound to Glutathione Sepharose 4B beads as described above and mixed with 1 µg/mL total RNA, mRNA, ssDNA or dsDNA purified from AGS cells in binding buffer (10 mM HEPES, pH7.4, 150 mM NaCl, 1 mM DTT, 0.01% Triton-X) for 20 min at 4 °C. PAR1b-FL was then added and further mixed for 1 h at 4°C. Beads were then washed four times with binding buffer, resolved on SDS-PAGE gel and stained with Coomassie Brilliant Blue for visualization.

### 4.5. Nucleic Acid Ligands

Total RNA from AGS cells was extracted with TRIzol Reagent (Life Technologies, Carlsbad, CA, USA) according to the manufacturer’s protocol. Purification of mRNA from AGS cells was performed using Oligotex-dT30 Super mRNA Purification Kit (TAKARA, Kusatsu, Japan) according to the manufacturer’s protocol. DNA purified from AGS cells was sonicated to 1000–3000 bp and extracted by phenol/chloroform. To make ssDNA, the dsDNA was boiled. Poly I:C HMW (InvivoGen, San Diego, CA, USA) was prepared according to the manufacturer’s protocol. Synthetic oligos were designed to the backbone of pBlueScript II KS (+) (Stratagene, La Jolla, CA, USA) for 20 nt (5′TATTGTCTCATGAGCGGATA3′) and 100 nt ssDNA (5′TATTGAAGCATTTATCAGGGTTATTGTCTCATGAGCGGATACATATTTGAATGTATTTAGAAAAATAAACAAATAGGGGTTCCGCGCACATTTCCCCGAA3′). 1000 nt ssDNA was prepared by boiling the PvuI-PvuI fragment of pBlueScript II KS (+). Linearized 3000 bp dsDNA was prepared by digesting the pBlueScript II KS (+) plasmid with EcoRV. 3000 nt ssDNA was prepared by boiling the 3000 bp dsDNA. Salmon sperm DNA (Sigma-Aldrich, St. Louis, MO, USA) was extracted by phenol and then phenol/chloroform, sonicated to 1000 bp–3000 bp and re-extracted by phenol/chloroform.

### 4.6. In Vitro Kinase Assay

PAR1b was incubated for 1 h at 30 °C with 200 µM TR1 peptide (NVKSKIGSTENLK) as a substrate in 50 mM Tris-Cl, pH7.5, 5 mM MgCl_2_, 2 mM EGTA, pH7.5, 0.5 mM DTT, 0.5 mM Benzamidine, 0.5 mM PMSF, 0.01% Triton X-100, 100 µM ATP in the presence of nucleic acid ligands. Production of ADP was quantified using the Fluorospark Kinase/ADP Multi Assay Kit (Fujifilm Wako, Osaka, Japan) according to the manufacturer’s protocol. An LS55 (Perkin Elmer, Waltham, MA, USA) fluorescence spectrometer was used for detection.

### 4.7. Mammalian Expression Vectors

The pEF expression vector for T7-PAR1b-FL and pSP65SRα expression vector for FLAG-PAR1b-FL have been described previously [21]. An expression vector for HA-PAR1b-FL was made by cloning in a sequence for the HA tag, generated by PCR, into pSP65SRα-FLAG-PAR1b-FL to replace the N-terminal FLAG sequence. Truncated fragments of PAR1b-N(1-331) and PAR1b-C(332-745) with an N-terminal FLAG sequence were generated by PCR and cloned into pSP65SRα. PAR1b-∆spacer was generated by excising the SacI-SacI fragment from pSP65SRα-FLAG-PAR1b-FL. This study uses the 745 aa isoform of PAR1b encoded by transcript variant 1 (GenBank: NM_017490.3) and therefore the amino acid residue numbers refer to the positions in this isoform. The pcDNA3.1 expression vector for FLAG-tau has been described previously [13] and expresses the 0N4R isoform of tau. An expression vector for HA-tau was made by cloning in a sequence for HA tag, generated by PCR, into pcDNA3.1-FLAG-tau to replace the N-terminal FLAG sequence. The pSUPER expression plasmid (Oligoengine, Seattle, WA, USA) for EBERs was made by PCR amplification of the genomic fragment containing EBERs 1 and 2, followed by insertion of the fragment downstream of the H1 promoter.

### 4.8. Cell Culture and Transfection

AGS human gastric epithelial cells were cultured in RPMI 1640 medium supplemented with 10% fetal bovine serum at 37 °C in 5% CO_2_. HEK293T human embryonic kidney cells were cultured in high glucose DMEM supplemented with 10% fetal bovine serum at 37 °C in 5% CO_2_. Cells were transfected with expression vectors, nucleic acid ligands, or PAR1b-specific siRNA (PAR1b-siRNA-535 [52]) using Lipofectamine 2000 reagent (Invitrogen, Waltham, MA, USA) according to the manufacturer’s protocol.

### 4.9. Generation of Stably Transfected Cell Lines

AGS cells were transfected with the linearized expression vector and linearized pBabe-puro at a ratio of 20:1 using the Lipofectamine 2000 reagent. 24 h after transfection, cells were passaged into medium containing 1 µg/mL puromycin, and clones were isolated either by cloning cylinders (EBERs) or by limiting dilution (HA-tau). Expression of EBERs was confirmed by EBER in situ hybridization and quantitative RT-PCR.

### 4.10. Immunoprecipitation and Immunoblotting

Immunoprecipitation and immunoblotting were performed as previously described [21]. For the RNase experiment, 10 µg/mL RNase A (DNase and protease-free; Thermo Fisher Scientific, Waltham, MA, USA) was added to the total cell lysate and incubated for 1.5 h at 4 °C prior to immunoprecipitation. Intensity of bands were quantified using the LAS-4000 Luminescent Image Analyzer System (Fujifilm, Tokyo, Japan).

### 4.11. DNA-Immunoprecipitation (DNA-IP)

A total of 1.5 × 10^6^ AGS cells were transfected with 15 µg of FLAG-PAR1b expression vectors complexed with 30 µL of Lipofectamine 2000. After 24 h, cells were subjected to a second round of transfection for 2 h with a mixture of 30 ng of pBlueScriptII KS (+) and 30 µg of salmon sperm dsDNA complexed with 30 µL of Lipofectamine 2000. DNA-IP was performed using the same protocol as immunoprecipitation except that in the final step, the beads were split into two. Immunoblotting was performed on one portion of beads and DNA was extracted from the other portion by phenol/chloroform followed by ethanol precipitation. The specific primers used to detect pBlueScript II KS (+) were For1 (5′GAACGTGGCGAGAAAGGAAG3′) and Rev1 (5′ACAGTTGCGCAGCCTGAATG3′). Quantitative PCR was performed using TB Green Premix Ex Taq (Tli RNaseH Plus; TAKARA, Kusatsu, Japan) according to the manufacturer’s protocol.

### 4.12. Statistics

Statistical analysis was performed using the GraphPad Prism Version 6.0 h (GraphPad Software, San Diego, CA, USA).

## Data Availability

Not applicable.

## Supplementary Materials

The following supporting information can be downloaded at: https://www.mdpi.com/article/10.3390/ijms23126634/s1.
