# Supplementary material for: Kinase Activity of PAR1b, Which Mediates Nuclear Translocation of the BRCA1 Tumor Suppressor, Is Potentiated by Nucleic Acid-Mediated PAR1b Multimerization
Source: Int J Mol Sci. 2022 Jun 14;23(12):6634. doi: 10.3390/ijms23126634 (PMC9223676; doi:10.3390/ijms23126634)
Supplement: Supplementary file 1 [file ijms-23-06634-s001.zip › ijms-1745665-supplementary.pdf]

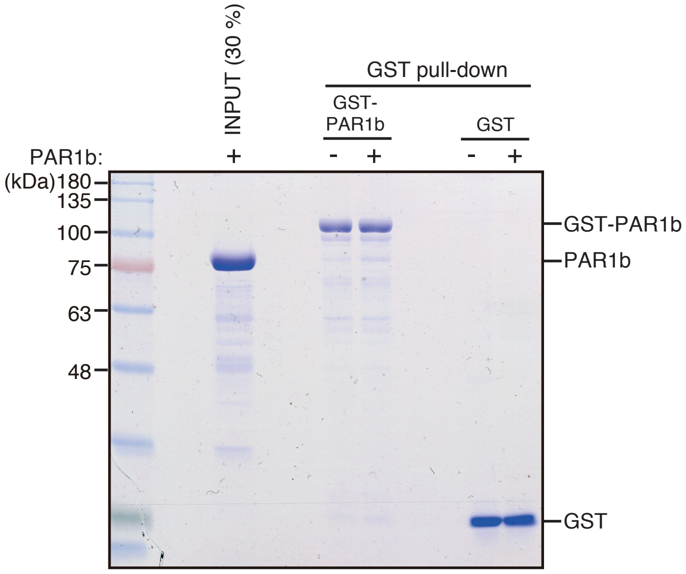

Figure S1. Recombinant full-length PAR1b does not multimerize spontaneously. GST-PAR1b bound to Glutathione Sepharose beads was mixed with 300 nM PAR1b for the GST pull-down assay.

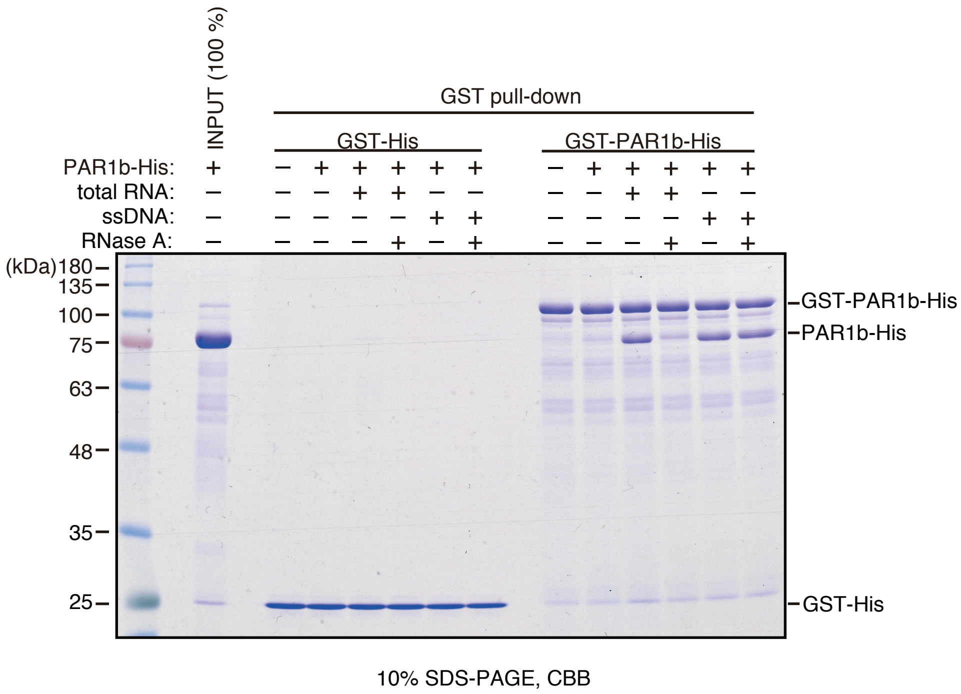

Figure S2. Both total RNA and ssDNA can mediate multimerization of PAR1b. GST-PAR1b bound to Glutathione Sepharose beads was mixed with 100 nM PAR1b in the presence of 1 μg/ml total RNA or ssDNA from AGS cells. Mixtures were treated with 10 μg/ml of RNase A to show that ssDNA was not contaminated with RNA.

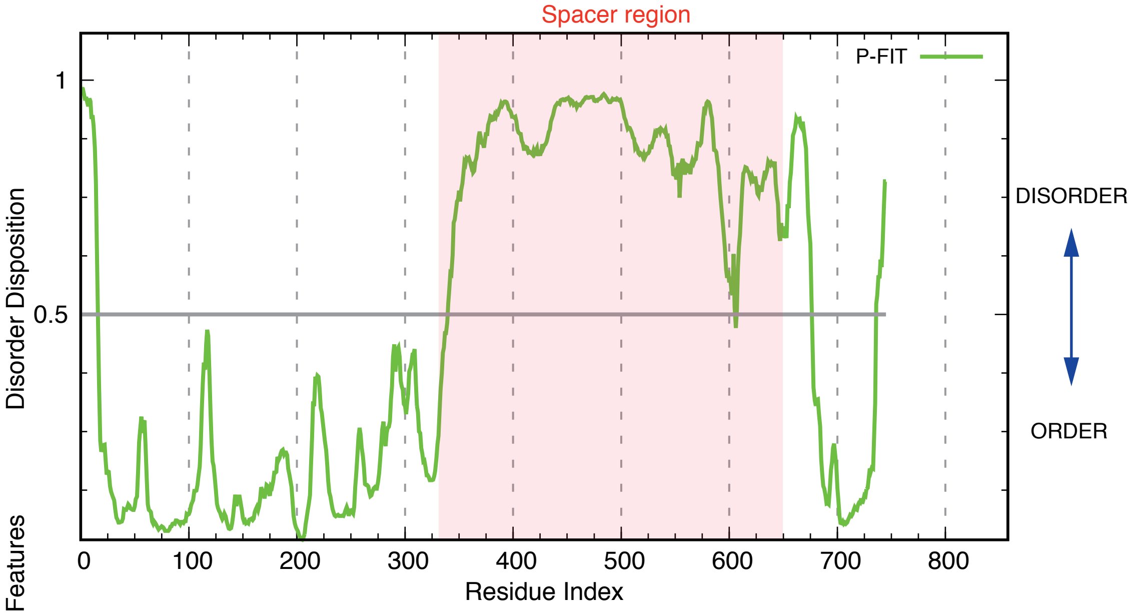

Figure S3. Spacer region of PAR1b is predicted to be intrinsically disordered.

Intrinsically disordered regions of PAR1b were predicted by the meta-disorder predictor PONDR-FIT. The gray line at 0.5 of the y-axis indicate the threshhold for disorder/structured residues. Residues above this line are predicted to be disordered while scores below 0.5 are predicted to be ordered.

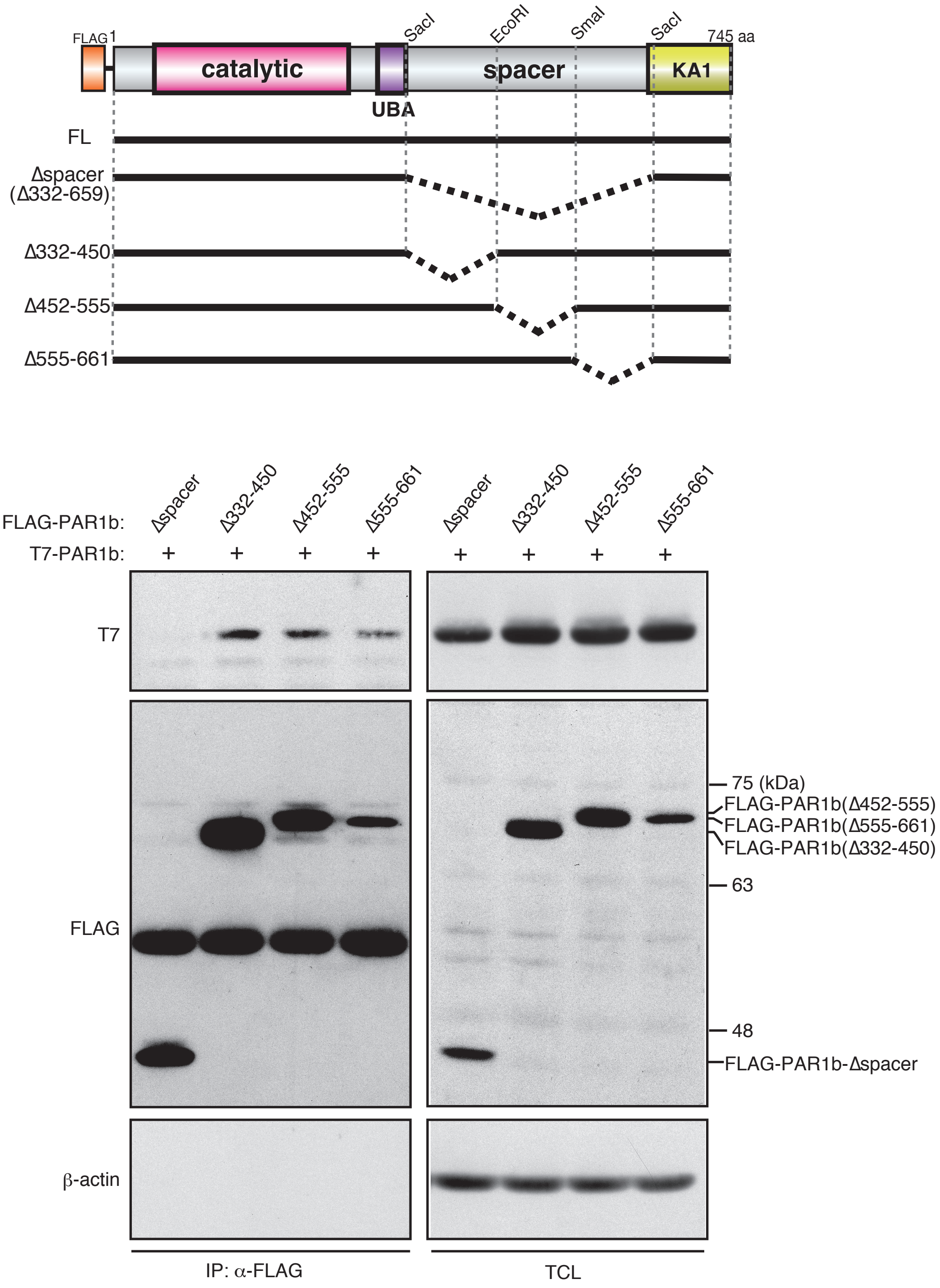

Figure S4. PAR1b with partial deletions in the spacer region can still form multimers. Schematic diagram of the series of deletions made in FLAG-PAR1b (*top*). AGS cells transiently transfected with expression vectors for T7-PAR1b and FLAG-PAR1b with partial deletions in the spacer region were subjected to immunoprecipitation (*bottom*).

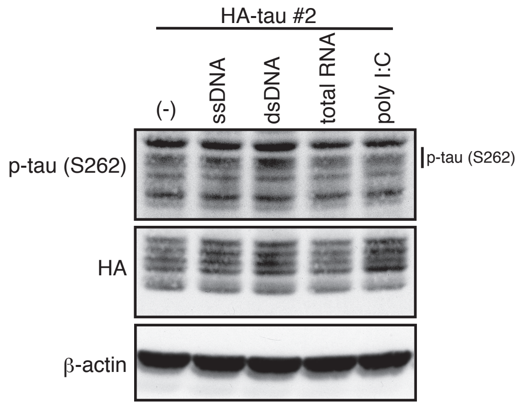

Figure S5. The effect of various nucleic acids on the phosphorylation of tau (S262) in AGS cells stably expressing HA-tau. A second stable cell line transfected with various nucleic acids confirm that dsDNA potentiates phosphorylation of tau at S262. ssDNA and dsDNA are from salmon sperm. Total RNA was purified from AGS cells.
